# Effects of Policy Intervention on Food System Resilience to Emergency Risk Shock: Experience from China during COVID-19 Pandemic

**DOI:** 10.3390/foods12122345

**Published:** 2023-06-11

**Authors:** Mingjie Cui, Xinhuan Zhang, Yufang Zhang, Degang Yang, Jinwei Huo, Fuqiang Xia

**Affiliations:** 1 State Key Laboratory of Desert and Oasis Ecology, Key Laboratory of Ecological Safety and Sustainable Development in Arid Lands, Xinjiang Institute of Ecology and Geography, Chinese Academy of Sciences, Urumqi 830011, China; 2 University of the Chinese Academy of Sciences, Beijing 100049, China

**Keywords:** lockdown policy, food security emergency policy, food price, Chinese experience, food-producing area, food-consuming area

## Abstract

Achieving the goal of zero hunger within the goal of sustainable development requires improving the resilience of food systems to various types of risk shocks; food systems have shown significant vulnerability to COVID-19 outbreaks and transmission. By analyzing the impact of China’s lockdown policy and food security emergency policy in 2020 on food prices during the COVID-19 pandemic, we can clarify the role of policy intervention in enhancing the resilience of the food system, which can provide guidance, using China’s experience, for dealing with global food safety emergencies in the future. Firstly, we selected Beijing, Shanghai and Guangdong as food-consuming areas, and Shandong, Henan, and Hubei as food-producing areas. We also collected food security emergency policy data from the Chinese government website during the COVID-19 pandemic. Secondly, a difference-in-difference method was used to observe that Chinese cabbage and pork prices in the main food-producing areas and food-consuming areas rose more obviously after the adoption of lockdown policy, and Chinese cabbage and pork prices in the food-consuming areas increased more obviously than those in food-producing areas. However, staple food prices have not risen significantly. Thirdly, the response of four kinds of food prices to the food security emergency policy is analyzed quantitatively and graphically using the food price volatility index and food price increase rate; we observed that the response of food prices to the food security emergency policy is related to the food types and regions. For food types, the fluctuation degree and increase in Chinese cabbage and pork prices decreased significantly after the adoption of the food security emergency policy. For regions, when the food security emergency policy was adopted, the food prices in the main food-consuming areas fluctuated more obviously than those in food-producing areas. Finally, we found that the implementation of the transport policy and the joint supply emergency policy in the main producing and consuming areas has played a very significant and positive role in stabilizing food prices.

## 1. Introduction

According to the 2022 Global Report on Food Crises of the World Food Programme (WFP), the number of people around the world facing food insecurity and in need of emergency food assistance and livelihood support has increased at an alarming rate [1]. In 2021, about 828 million people were affected by hunger, accounting the 9.8% of the world’s population. Furthermore, the number of people facing or suffering from severe food insecurity increased from 130 million to 345 million from 2019 to 2021. Almost 40 million people across 36 countries were facing conditions of emergency or worse (IPC/CH Phase 4 or above). More than half a million people were facing catastrophe conditions, including starvation and death (IPC/CH Phase 5) in Ethiopia, South Sudan, Southern Madagascar, and Yemen. The number of people facing this condition in 2021 is four times that in 2020 and seven times that in 2016 in these four countries. Due to the increasing frequency and severity of extreme weather, the outbreak of the COVID-19 pandemic [2], increasing conflict and insecurity [3], and increases in global food prices [4], global and local food systems are under increasing pressure. The food system is vulnerable to various kinds of risk shocks [2]. Progress in achieving the SDG 2 zero hunger goal (SDGs) proposed by the United Nations is affected by these shocks. Sustainable development requires accelerating the resilience of the food system to various risk or shocks.

Since December 2019, the impact of COVID-19 on food systems has been significant [5], and researching the impact of such public health emergencies on food systems can help us summarize the experience of the resilience of food system. From a macro perspective, COVID-19 has triggered a global economic recession and agricultural trade shrinking, influencing the global food supply chain and causing an increase in food prices. The instability, imbalance, and vulnerability of the global food system have become more significant. From a micro perspective, lockdown policies in response to COVID-19 have impacted the food system in terms of production, circulation, consumption, and other aspects. Hence, changes in food prices are one of the most important indicators of the food system’s stability.

Food prices during the COVID-19 pandemic have become an important topic for interested scholars. Some scholars have predicted an increase of food prices in the short term due to storage patterns and hoarding purchases, from a global perspective [6,7]. Some scholars have studied changes in food prices during the COVID-19 pandemic in individual countries. Bairagi et al. [8] analyzed price changes in storable and perishable foods in India, and found that wheat flour and rice prices increased significantly, while onion prices decreased significantly. Çakır [9] analyzed wholesale fruit and vegetable prices in the US and China, and found that the wholesale price of fruits and vegetables in China was falling, while the wholesale price of fruits and vegetables in the US varied non-significantly. Bai et al. [10] compared monthly retail food prices in up to 181 countries from January 2019 to June 2021, and found that the price of more nutritious food groups in countries rose significantly with higher COVID-19 cases counts. Akter et al. [11] found that food access was more difficult and food price increased after the advent of COVID-19 compared to pre-COVID-19 in Bangladesh. These studies show that food prices not only vary from country to country, but also vary with different food types when affected by COVID-19. Some scholars have studied changes in food prices during the pandemic at a local level. Yu et al. [12] found that the Chinese cabbage price in Beijing and Shandong showed an increasing trend, but the Chinese cabbage price in Hubei showed a decreasing trend, and that the wholesale prices of wheat flour and rice in Beijing, Hubei, and Shandong did not change significantly. These studies demonstrate that changes in food prices are related to regional development and food types.

Furthermore, governments have taken preventive measures (such as closing border crossings, schools, workplaces, businesses, international travel restrictions, etc., called ‘lockdown policies’) because of the COVID-19 pandemic. This lockdown policy has become an effective method of restricting the movement of people in a country and containing the spread of the virus, but it has proved to be an important reason for rising food prices. As a result, there are a large number of studies that empirically examine the impact of lockdown policies on food prices [13]. They found that food prices change differently in different cities. Narayanan et al. [14] found that food prices in cities rise significantly after lockdown, and food prices in small cities rise more than those in big cities. Other research has proved that different food types also show great heterogeneity before and during lockdowns, and that perishable food prices of items such as meat, fruits, and vegetables increased more obviously [15]. Moreover, there is regional heterogeneity in food price changes. Tomato prices increased significantly during the lockdown compared with the pre-lockdown period in the states of Maharashtra, Jharkhand, and Meghalaya, India [16], while tomato prices decreased significantly in Haryana, India [17]. Other scholars have analyzed the impact of specific policies on food prices, and found that the severity of the implementation of stay-at-home restrictions has an impact on food prices, and that prices in food categories such as meat, fish, seafood, and vegetables increase significantly [18]. The above studies either compared food prices before and during the lockdown or conducted quantitative regression analyses of the relationship between lockdown policy and food prices to show the impact of lockdown policy on food prices. At present, there are few studies that analyze the impact of food safety policies on food prices during the COVID-19 lockdown. Although Jiang et al. [19] compared the food security policies of China, Italy, Malawi, and Argentina, this study only analyzed the food security policies among countries, and did not analyze the impact of food security policies on the food prices.

In general, these existing studies have shown that the implementation of lockdown policies during the COVID-19 pandemic has a significant impact on food prices. However, no studies have looked at the relationship between food security emergency policies and food prices during the COVID-19 pandemic. In China, a series of policies to ensure food safety were implemented when lockdown policies came into force during the COVID-19 pandemic. Therefore, compared with previous studies, this paper focuses on the relationship between food security emergency policies implemented during the COVID-19 pandemic and food prices. A quantitative and graphical analysis of the effects of China’s food intervention policies on food prices during the COVID-19 pandemic, through the two indexes of the food price volatility index (*CV*) and food price increase rate (*ROC*), can fill in the gap existing in empirical research on the impact of COVID-19 on food prices, and will also provide guidance, using China’s experience, for improving the global food safety policy system.

Lastly, the period of the most severe epidemic in China (January 2020 to October 2020) is selected for this research, because it reflects the main epidemic intervention policies in China during this period. Although China’s epidemic intervention policies have changed (largely after December 2022), researching the impact of food intervention policies on prices during the period (January 2020 to October 2020) of the most severe epidemic in China means our results are highly representative.

Section 2 describes the data and method. Section 3 presents descriptive statistics and results, and Section 4 discusses the reasons for the results. Section 5 summarizes the results of Section 3 and Section 4, and offers concluding remarks.

## 2. Materials and Methods

### 2.1. Study Area and Lockdown Time

The per capita food expenditure of Beijing, Shanghai, and Guangdong is far higher than the national average level. The per capita food consumption expenditure levels of Beijing, Shanghai, and Guangdong are ranked sixth, first and second, respectively, in China. They are famous areas of consumption in China. Shandong, Henan, and Hubei are China’s major grain production, vegetable planting, and meat production and processing bases. They are famous agricultural provinces in China. In this study, the main food-consuming areas of Beijing, Shanghai, and Guangdong, and the main food-producing areas of Shandong, Henan, and Hubei were selected as the study areas; Hubei was also the area most affected by COVID-19.

Since there is no clear lockdown time for other provinces, the starting time of the lockdown was chosen according to the time at which Beijing, Shanghai, Guangdong, Hubei, Shandong, and Henan started the first level of response [20]. Since the whole country gradually resumed work and production on 5 March 2020, and the state of total lockdown basically ended, this date is taken as the ending time of the lockdown. The lockdown time of Hubei province was calculated according to the lockdown starting time and the lockdown ending time of Wuhan City. The starting and ending times of the lockdown in all provinces are shown in Table 1. below. Additionally, the lockdown periods of the main food-consuming areas and main food-producing areas were calculated according to the earliest lockdown time and the latest ending time of lockdown (the ending time of the lockdown in the main food-producing areas is calculated according to Shandong and Henan province). Therefore, the lockdown period for the main food-consuming areas and food-producing areas was taken to be 23 January 2020 to 5 March 2020. The lockdown period in Hubei province was 23 January 2020 to 8 April 2020.

### 2.2. Food Type

According to Chinese dietary and consumption habits, Chinese cabbage, pork, flour, and rice were selected as the four food types discussed in this article. Chinese cabbage is grown in a wide range of areas in China, accounting for about 15% of the country’s vegetable planting area, and the per capita consumption of Chinese cabbage accounts for about 60% of vegetable consumption; therefore, this commodity plays an important role in balancing market supply and stabilizing market prices [21]. Pork is dominant in China’s meat market [22]; pork production and consumption accounted for about 63.4% and 62.9% of the meat market, respectively, in 2018 [23]. Staple food consumption in China is mainly made up of flour and rice, due to the historic eating habits of Chinese people. Therefore, these four kinds of food generally represent the types of food consumed in China.

### 2.3. Food Security Policies

During the COVID-19 pandemic, China’s food supply was affected by three kinds of policies. One is the grain reserve policy, including state grain reserves and local grain reserves. This refers to the grain and edible oil reserved by the central government for long-term adjustment of the total supply of and demand for grain in the country, thereby stabilizing the grain market and coping with major natural disasters or other emergencies in the long term. It is an important material means for the state to regulate the grain market, and grains are an important strategic material related to national economic security. Another policy is the meat reserve policy, including state reserves and local reserves. This includes the live pig reserve and frozen meat reserve set up by the Ministry of Commerce in all provinces and cities of the country, and includes the meat products reserved by the state in order to cope with abnormal market fluctuations and regulations caused by major natural disasters, public health events, animal epidemics, or other emergencies. The state grain reserve policy and state meat reserve policy are both long-term national macro-control policies. Another policy is the interim policy (called the ‘food security emergency policy’ in this article), used to guide food supply and price stabilization during the lockdown. We collected the food security emergency policies used from 23 January 2020 to 25 February 2020 from the government’s official website; the policies relate to the supply of daily necessities within the epidemic prevention and control policies, and we divided them into different types of food security emergency policies (Table 2.).

### 2.4. Methods

#### 2.4.1. DID Model

The differences-in-difference (referred to as the “DID”) method is mainly used in sociology to evaluate the effects of policies. This method estimates the effect of policy intervention using the difference between the intervention group and the control group before and after the intervention (judged based on the coefficient of the intersection term). This article is based on the lockdown policies of the epidemic; food prices were set for the control group, using those from 1 January 2019 to 30 April 2019. Food prices were set for the intervention group, using those from 1 January 2020 to 30 April 2020. The analysis was carried out by comparing the differences before and during the lockdown. More specifically,

(1) $$Y_{it}=\alpha_{0}+\alpha_{1}d_{t}+\alpha_{2}d_{g}+\alpha_{3}d_{t}\times d_{g}+\varepsilon_{it}$$

In Equation (1), *Y_it_* is the food prices of the explanatory variable, *d_t_* is the time dummy variable, if *d_t_* = 0 indicates that before the lockdown policy is implemented and *d_t_* = 1 indicates that after the lockdown policy is implemented. *d_g_* is the group control variable if *d_g_* = 0 indicates no lockdown policy intervention and *d_g_* = 1 indicates lockdown policy intervention. *d_t_* × *d_g_* is the cross term, namely the product of *d_t_*, and *d_g_*. *α*_1_ *α*_2_ *α*_3_ are the coefficients, respectively, where *α*_3_ is the cross-term coefficient, representing the DID processing effect. When it passes the significance test, the difference is significant, which indicates that prices changed significantly before and during the implementation of the lockdown policy. If *α*_3_ > 0, a positive correlation is indicated, and during the implementation of the lockdown policy, an increase in food prices will be promoted; if *α*_3_ < 0, a negative correlation is indicated, and during the implementation of the lockdown policy, prices will reduce. *ε_it_* is the random disturbance term.

The difference-in-difference method (DID) was used to conduct a regression analysis of daily food prices before and during the lockdown, revealing the difference in the effect of lockdown policies on four kinds of food prices.

#### 2.4.2. Price Volatility Index

In this paper, the coefficient of variance (*CV*) is used to represent the food price volatility index. The coefficient of variance, also known as the “standard margin” or “unit risk”, is a statistic used to measure the degree of variation in each observed value in the data; it reflects the degree of dispersion on the unit mean. The coefficient of variance is the ratio of the variance index of a set of data to its average index. It is a relative variation index, commonly used as the coefficient of standard deviation, expressed by *CV*. More specifically,

(2) CV=σ/μ

In Equation (2), *CV* represents the dispersion degree of food prices, namely the price volatility index. *σ* represents the standard deviation of food prices, and *μ* represents the mean of food prices. It can be seen from Equation (2) that the *CV* represents the proportion of the amplitude of data fluctuation along the mean value, and it is a measure of relative volatility. A higher *CV* means greater price volatility and more volatile prices.

In this paper, from 1 January 2020 to 30 April 2020, according to the time nodes of different food security emergency policies issued by the state department, the food price volatility indexes of the main food-producing areas and food-consuming areas before lockdown, during lockdown, and after lockdown were calculated, respectively, and the changes in the food price volatility indexes in the three different periods were observed. Considering that there was a slight difference during the lockdown between the food-consuming areas and food-producing areas, and cases began to appear in all provinces from the beginning of January 2020, a level of basically zero was accomplished in April. The pre-lockdown time of the main food-consuming areas in this paper is from 1 January 2020 to 22 January 2020, the lockdown time is from 23 January 2020 to 5 March 2020, and lifting of lockdown time is from 6 March 2020 to 30 April 2020. Similarly, the pre-lockdown time of the main food-producing areas in this paper is from 1 January 2020 to 22 January 2020, the lockdown time is from 23 January 2020 to 5 March 2020 (the lockdown time of Hubei is from 23 January 2020 to 8 April 2020), and the lifting of lockdown time is from 6 March 2020 to 30 April 2020 (the lifting of lockdown time in Hubei is from 9 April 2020 to 30 April 2020). Since COVID-19 is a public health emergency, in this paper, comparing and analyzing the food price volatility index when food security emergency policies are not adopted in time with the food price volatility index after food security emergency policies are adopted will reflect the influence of the food security emergency policies on the stabilization of food prices.

#### 2.4.3. Price Increase Rate

In this paper, the rate of change is used to represent the food price increase rate. The rate of change is the rate at which food prices increase or decrease, as expressed by *ROC*; it is a momentum indicator that measures the percentage of price increase from one period to the next period. More specifically,

(3) $$ROC=\frac{{(Fp}_{t}-{Fp}_{t-1})}{{Fp}_{t-1}}\times 100\%$$

In Equation (3), *ROC* is the price increase rate, *Fp_t_* is the food price in period t, and *Fp_t-1_* is the food price in period t-1. *ROC* > 0 indicates that food prices rise, and when the *ROC* value is larger, the food price increase is greater. *ROC* < 0 indicates that food prices fall, and when the *ROC* value is larger, the food price decrease is greater. *ROC* = 0 means that food prices are neither rising nor falling.

According to the time node of the promulgate of the food security policies and the time series change in food prices, the Chinese cabbage and pork price changes are divided into four stages in this paper, and the price change was observed at the start and end of each time period. We calculated the price increase rate of each stage, and compared the food price volatility index and food price increase rate values of each stage to clarify how the food price changes were affected by food security emergency policies during the lockdown period.

## 3. Results

In Section 3, the results are described in two parts. Section 3.1 is the operation result of the DID model, which analyzes the changes in Chinese cabbage prices, pork prices, and staple food prices before, during, and after lockdown, so as to clarify the impact of lockdown policies adopted during the COVID-19 pandemic on the prices of these three categories of food. Section 3.2 is the graphic results, found using the food price volatility index and the food price increase rate, which analyze how food prices fluctuated and increased during the lockdown. In Section 3.2, Section 3.2.1, Section 3.2.2, and Section 3.2.3, we present the results of the price analysis of Chinese cabbage, pork, and staple foods, respectively.

### 3.1. Food Price Changes before, during, and When Lifting Lockdown

For the Chinese cabbage and pork prices, the policy effect coefficients of the lockdown policies in the study areas all passed the positive 1% significance test (column 3 is the coefficient, and column 5 is the significance levels in Table 3). Among them, the policy effect coefficient (α_3_) of the Chinese cabbage price in the main food-consuming areas (Beijing, Shanghai, Guangdong) was between 0.1663 and 0.2927, indicating that the Chinese cabbage price increased by around 16% to 29% after the implementation of the lockdown policy. The policy effect coefficient (α_3_) of pork prices in the main food-consuming areas (Guangdong data missing) was between 1.8001 and 1.9719, indicating that pork prices increased by 180% to 197% after the implementation of the lockdown policy. The policy effect coefficient (α_3_) of the Chinese cabbage price in the main food-producing areas (Hubei, Shandong, and Henan) was between 0.4172 and 0.5169, indicating that the Chinese cabbage price increased by about 40% to 50% after the implementation of the lockdown policy. The policy effect coefficient (α_3_) of pork prices in the main food-producing areas was between 1.7032 and 2.0492, indicating that pork prices increased by 170% to 200% after the implementation of the policy. The above results indicate that the implementation of lockdown policy will cause Chinese cabbage and pork prices to rise, and the pork price rise is more obvious than that of Chinese cabbage.

As for staple foods, staple food prices in the main food-producing areas and main food-consuming areas were less affected by the lockdown policy. In the main food-consuming areas, the policy effect coefficient (α_3_) of staple food prices in Beijing and the flour price in Shanghai (staple food price data in Guangdong were missing, and the rice price in Shanghai did not pass the significance test) passed the positive 1% significance test; however, the coefficient was small, indicating that the price increase was less than 1% after the implementation of the lockdown policy. On the contrary, in the main food-producing areas, the policy effect coefficient (α_3_) of staple food prices in Henan and Shandong was small, and it did not pass the significance test, indicating that the staple food price change was not statistically significant and the lockdown policy had no significant impact on staple food prices after the implementation of the lockdown policy. The difference is that although Hubei is one of the main food-producing areas, the policy effect coefficient (α_3_) of staple food prices passed the 1% positive significance test; however, the coefficients were small, indicating that the staple food prices rose about 3% and 1%, respectively, after the implementation of the lockdown policy in Hubei province.

### 3.2. Impact of Food Security Policies on Four Kinds of Food Prices

#### 3.2.1. Chinese Cabbage Price Changes

**Figure 1:**
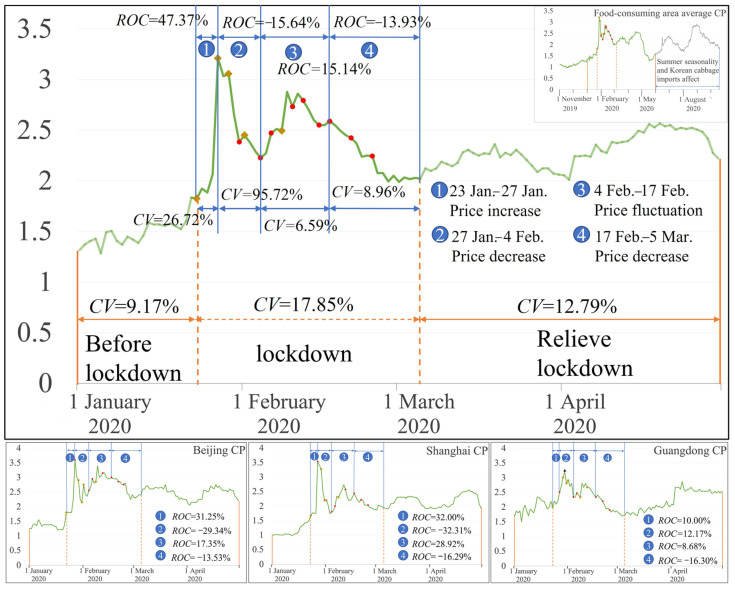
Chinese cabbage price changes in food-consuming areas during the lockdown (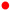 indicates food supply policies. 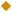 indicates food transport policies).

For the main food-consuming areas (Figure 1), the Chinese cabbage average price volatility index was 9.17%, 17.85%, and 12.79%, when before lockdown, during lockdown, and when lifting lockdown, respectively, which also reflected that the food price fluctuation was higher during lockdown than before lockdown and when lifting lockdown. Otherwise, *CV* was 26.72%, 95.72%, 6.59%, and 8.96% in the stage 1, stage 2, stage 3 and stage 4, respectively. The Chinese cabbage price rose between min-max prices during the lockdown, leading to an increase in the price volatility index (*CV*). However, *CV* did not decrease with more time points, and *CV* gradually fluctuated with more time points; it was significantly smaller than in stage 1 and stage 2. The Chinese cabbage price increased in the main food-consuming areas, and the Chinese cabbage price increase rate (*ROC*) was 47.37% in the early stage of the lockdown (23 January to 27 January). With the implementation of the food security emergency policies, including a food supply policy, on January 27 and a food transportation policy on 29 January, the Chinese cabbage price dropped sharply, showing a decrease of 15.64% compared to the early stage of the lockdown. In the following two weeks (4 February to 17 February), the Chinese cabbage price fluctuated and increased by 15.14% compared to the previous stage. After 17 February, with the implementation of the food security policy to ensure the stable production and supply of agricultural products and the continuous improvement of the multi-provincial co-guarantee and supply mechanism, the Chinese cabbage price continued to decline from 17 February to 5 March, by about 13.93%, compared to the third period. After relieving the lockdown, the circulation of Chinese cabbage gradually recovered, and the price volatility was significantly affected by market demand; the price volatility index dropped by 12.79% compared to the lockdown period.

As can be seen from the changing trends in Chinese cabbage prices in Beijing and Shanghai (Beijing CP, Shanghai CP in Figure 1), food security emergency policies play an important role in guiding food price stability. The Chinese cabbage price in Beijing and Shanghai rose sharply in the first stage of the lockdown (23 January to 27 January), with an increase of 31.25% and 32.00%, respectively, compared to before the lockdown; however, with the implementation of the food security emergency policy, it showed a rapid downward trend, decreasing by about 29.34% and 32.31%, respectively, compared to the first stage. However, the Chinese cabbage price changes in Guangdong are complicated (Guangdong CP in Figure 1); with the implementation of the food security emergency policy, the Chinese cabbage price increase gradually slowed down. After 17 February, the Chinese cabbage price in Guangdong dropped by about 16.3% compared to the third stage.

**Figure 2:**
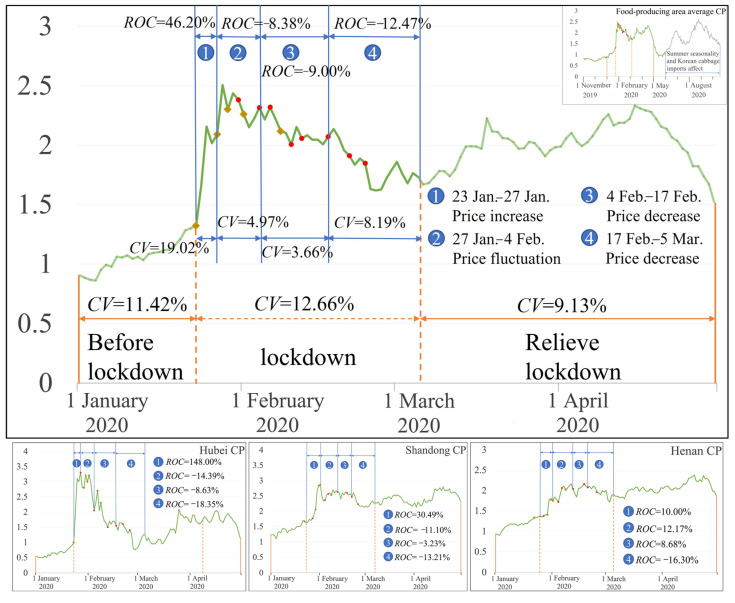
Chinese cabbage price changes in the food-producing areas (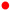 indicates food security policies. 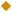 indicates food transport policies).

From the main food-producing areas (Figure 2), the Chinese cabbage average price volatility index was 11.42%, 12.66%, and 9.13% before lockdown, during lockdown and when lifting lockdown, respectively, which reflected that the Chinese cabbage price increased significantly because of the max price rise; however, the range of the increase was smaller than that of the main food-consuming areas in the same period. *CV* was 19.02%, 4.97%, 3.66%, and 8.19% in the stage 1, stage 2, stage 3 and stage 4, respectively, and *CV* decreased gradually compared to *CV* in stage 1; it was also significantly smaller than that in stage 1. The Chinese cabbage price change results can be divided into four stages (the first stage: 23 January to 27 January, the second stage: 27 January to 4 February, the third stage: 4 February to 17 February, and the fourth stage: 17 February to 5 March). In the first stage of the lockdown, the Chinese cabbage price increased by 46.2%. With the implementation of the food security emergency policy, including a food transportation policy and a food supply policy, from 29 January to 4 February, the Chinese cabbage price fluctuated, and the supply and demand of the Chinese cabbage market were adjusted. The Chinese cabbage price decreased by 8.38% compared with the first stage of the lockdown. With the continuous implementation of the food security emergency policy after 4 February, the Chinese cabbage price gradually dropped; after 17 February, the Chinese cabbage price dropped significantly, by about 12.47%, compared to the third stage.

From the trends in Chinese cabbage price change in Hubei, Shandong, and Henan (Hubei CP, Shandong CP, and Henan CP in Figure 2), we can observe that the influence of the food security emergency policy on food prices is different. In the first stage of the lockdown (23 January to 27 January), the Chinese cabbage price in Shandong and Henan increased significantly. From 17 to 27 January, with the intervention of the food security emergency policy, the Chinese cabbage prices in Shandong and Henan fluctuated slightly. After 17 February, the Chinese cabbage prices in Shandong and Henan showed a sharp decline. Hubei province is the most affected province in China, so its food price under the food security emergency policy intervention dropped the most. After 27 January, the Chinese cabbage price continued to decline, and the biggest drop saw prices reach a level lower than that before the lockdown.

#### 3.2.2. Pork Price Changes

**Figure 3:**
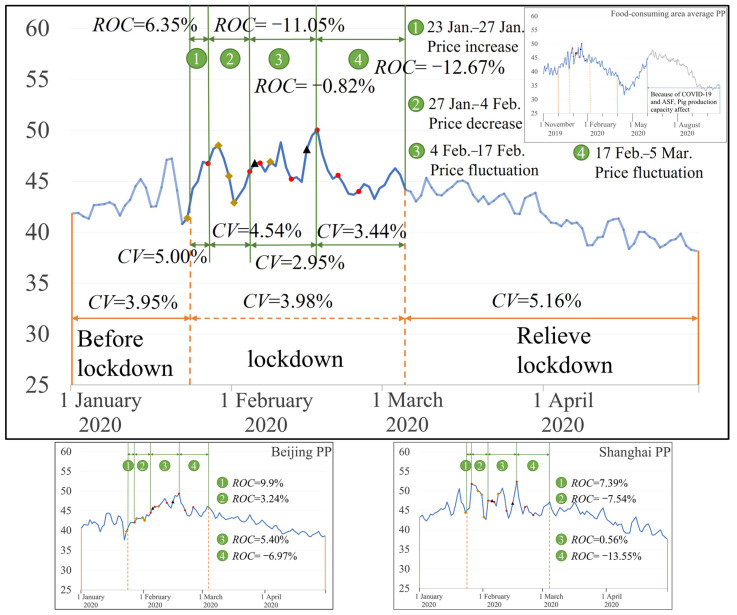
Pork price changes in the food-consuming areas (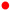 indicates food security policies. 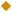 indicates food transport policies. 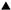 indicates meat supply policies).

For the main food-consuming areas (Figure 3), the pork average price volatility index was 3.95%, 3.98%, and 5.16% before lockdown, during lockdown, and when lifting lockdown, respectively, indicating that the fluctuation degree of pork prices during lockdown was higher than before lockdown and lower than when lifting lockdown. *CV* was 5.00%, 4.54%, 2.95%, and 3.44% in the stage 1, stage 2, stage 3 and stage 4, respectively, and *CV* decreased gradually compared to *CV* in the stage 1. The pork price changes can be divided into four stages (the first stage: 23 January to 27 January, the second stage: 27 January to 4 February, the third stage: 4 February to 17 February, the fourth stage: 17 February to 5 March). In the early stage of the lockdown (23 January to 27 January), the pork prices gradually rose by 6.35%. In the next week, food transportation policies continued to be implemented on 29 January, 31 January, and 1 February to ensure the smooth transportation of pork during the lockdown, and pork prices dropped by 11.05% compared to the first period. Then, within two weeks (4 February to 17 February), due to the implementation of food security policies such as food supply policies, transportation policies and policies for the resumption of livestock production, the state released 30,000 tons of central reserve meat [24], which effectively regulated the supply and demand of pork market. After 17 February, pork prices fell sharply, by 12.67%, compared to the third period.

Specifically (Beijing PP and Shanghai PP in Figure 3), the response of pork prices to food security emergency policies in Beijing and Shanghai was different. In Beijing, pork prices continued to rise from 23 January to 17 February, and the maximum increase reached 24.21% from the day before the lockdown. After 17 February, pork prices fell, and decreased by 6.97% compared to the third stage. The fluctuation of pork prices in Shanghai can be roughly divided into four stages. Pork prices gradually rose in the first stage, increasing by about 7.39% compared to before lockdown. In the second stage, pork prices gradually decreased. In the third stage, the pork price fluctuated and increases slightly, increasing by about 0.56% compared to the previous period. In the fourth stage, pork prices fell sharply, almost back to the level of the day before the lockdown. Therefore, after 17 February, pork prices in Beijing and Shanghai gradually decreased, and pork prices did not fluctuate significantly afterward, which indicates that the multi-regional co-guarantee and joint supply policy had an obvious effect on the stability of pork prices.

**Figure 4:**
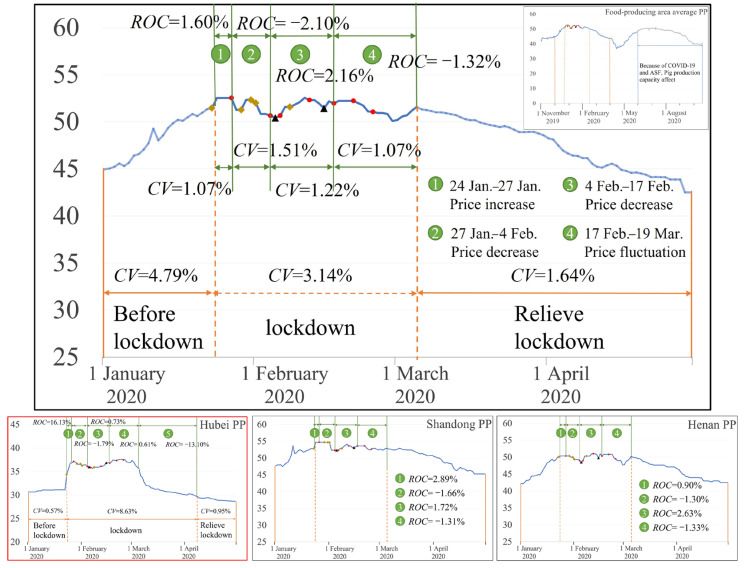
Pork price changes in the food-producing areas (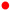 indicates food security policies. 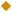 indicates food transport policies. 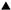 indicates meat supply policies).

For the main food-producing areas (Figure 4), the pork price volatility index was 4.79%, 3.14%, and 1.64% before lockdown, during lockdown and when lifting lockdown, respectively, indicating that the fluctuation of pork prices was small during the lockdown period, even lower than the level before the lockdown period. *CV* was 1.07%, 1.51%, 1.22%, and 1.07% in stage 1, stage 2, stage 3 and stage 4, respectively, and *CV* did not change much with more time points. This further indicates that the food security emergency policy adopted during the lockdown period had a significant positive effect on the stability of pork prices. The pork price increase rate in the main food-producing areas was small compared to the day before the lockdown, and the maximum increase in pork price was not more than 2.5%. Pork prices in the food-producing areas rose much less than in the food-consuming areas, indicating that pork prices in main food-consuming areas are more likely to undergo fluctuations in response to public health emergencies. The graph results for Shandong and Henan provinces (Shandong PP and Henan PP in Figure 4) show that the pork price did not fluctuate and increase significantly, similar to the average pork price in the main food-producing areas.

Although Hubei province is one of the main pork-producing areas, it is also a severely affected area. For the pork price in Hubei (Hubei PP in Figure 4), the pork price volatility index was 0.57%, 8.63%, and 0.95% before lockdown, during lockdown and when lifting lockdown, respectively, indicating that the fluctuation in pork prices in Hubei province during lockdown was larger than that before lockdown and when lifting lockdown. Furthermore, the pork price rose significantly in Hubei province from 23 to 27 January (first stage), increasing by about 16.13% compared with the day before the lockdown. From 27 January to 1 March, due to the active assistance among provinces, the Hubei pork supply was sufficient, and the pork price did not fluctuate significantly. However, the pork price remained high, and increased by 1.55% compared with the first stage of the lockdown. From March onwards, Hubei province increased its supply of reserve meat products [25], and pork prices dropped significantly. Therefore, China’s long-term national meat reserve policy has been proven to effectively stabilize pork prices when dealing with a public health emergency.

#### 3.2.3. Staple Food Prices Changes

**Figure 5:**
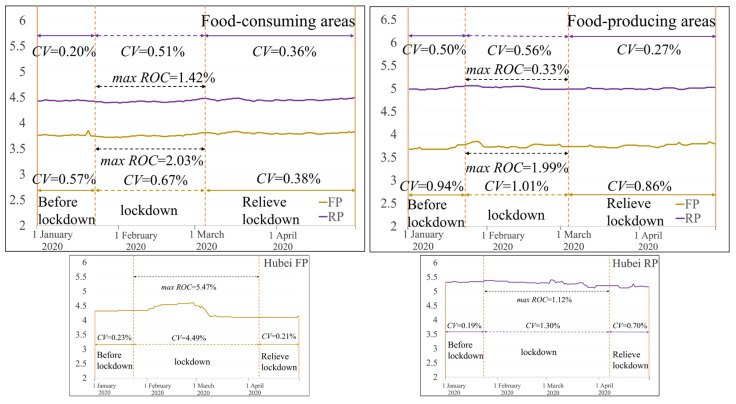
Staple food price changes in food-consuming and -producing areas.

Overall, for staple food prices including flour price and rice price in the main food-consuming areas (food-consuming areas in Figure 5), the flour price volatility index was 0.57%, 0.67%, and 0.38% before lockdown, during lockdown and when lifting lockdown, and the rice price volatility index was 0.20%, 0.51%, and 0.36% before lockdown, during lockdown and when lifting lockdown, respectively. The staple food prices maximum increased about 2.03% and 1.42%, respectively compared to the day before lockdown. For staple food price in the main food-producing areas (food-producing areas in Figure 5), the flour price volatility index was 0.94%, 1.01%, and 0.86% before lockdown, during lockdown and when lifting lockdown, and the rice price volatility index was 0.50%, 0.56%, and 0.27% before lockdown, during lockdown and when lifting lockdown, respectively; the staple foods’ maximum price increased by about 1.99% and 0.33%, respectively, compared to the day before lockdown. Thus, during the lockdown period, the fluctuation range of staple food prices in the producing and consuming areas was small, and did not exceed 1.01%. Although the staple food prices increased somewhat, the maximum increase was no more than 2.03%. Compared with the volatility index and the increase rate of the Chinese cabbage and pork prices, the volatility index and the increase rate of the staple food prices varied minimally.

Observing the changes in staple food prices in Hubei (Hubei FP, Hubei RP in Figure 5), the flour price volatility index in Hubei was 0.23%, 4.49%, and 0.21% before lockdown, during lockdown and when lifting lockdown, respectively, and the rice price volatility index in Hubei was 0.19%, 1.30% and 0.70% before lockdown, during lockdown and when lifting lockdown, respectively. This indicated that the price fluctuation degree of flour in Hubei province was large during the lockdown period, and the flour price volatility index was larger than that of rice price. In addition, the flour price in Hubei rose by a large amount, with a maximum increase of 5.47% compared with the day before the lockdown, while the rice price rose by a small amount, with a maximum increase of 1.12% compared with the day before the lockdown.

## 4. Discussion

In responding to a public health emergency, the food prices reacted to food security policies differently, depending on the different food types and regions. On the one hand, in terms of food types, vegetable price fluctuations and increases are the highest after the implementation of lockdown policies; these are followed by meat prices and then staple food prices, as confirmed by this paper and other studies [12]. After the implementation of food security policies, the degree of fluctuation and increase in vegetable and meat prices noticeably decreased. Such differences are mainly reflected in the storage capacity and supply chain of different foods, as well as the implementation of national policies and interventions. Vegetables are perishable and difficult to store, and their supply chain faces significant supply and demand conflicts under lockdown conditions, both during transportation and consumption. When food security emergency policies such as food supply policies, food transportation policies, and meat supply policies are implemented, the transportation and supply problems of vegetables are effectively resolved, and vegetable prices decrease significantly. Meat is more storable than vegetables and the government has a certain amount of meat reserved for the long term. So, with the timely release of reserved meat, the implementation of food security emergency policies, and the resumption of meat production by enterprises during the lockdown, the meat supply gradually increased. Additionally, due to a decrease in demand caused by less frequent purchases by consumers [12], the supply and demand of meat gradually balanced, and the price fluctuation and increase gradually decreased. Staple foods have a higher storage capacity than vegetables and meat, and China attaches great importance to the storage and supply of staple foods, having long-term policies for central, local, and emergency reserve grain storage. During the epidemic, there were ample reserves of staple foods [12], and the government could release the reserve grain based on the market supply and demand, ensuring the demand of the grain market, even in the context of large-scale purchasing. On the other hand, in terms of regional heterogeneity, during lockdown periods, after the implementation of food security emergency policies, food prices in the main consuming areas fluctuated and increased more significantly than those in the main producing areas. This is mainly due to the distance between food production and consumption [26], and transportation capacity. In the main consuming areas, the food production and processing capacity is limited, and some large and medium-sized cities even rely entirely on inter-regional transportation. During this epidemic, long-distance interregional transportation and supply were severely affected [27]. After implementing food security emergency policies such as establishing material transfer stations and carrying out multi-regional cooperation in production and sales areas, the food supply chain in the main consuming areas was effectively repaired, and the transportation capacity between regions was improved, thereby stabilizing food prices in the main consuming areas. In contrast, in the main producing areas, food production and reserves are relatively sufficient, and food transportation between cities in the main producing areas is less affected by lockdown. Compared to the main consuming areas, the main producing areas have advantages in food production, reserves, and transportation.

In response to public health emergencies, timely adoption of food security emergency policies can stabilize food prices and effectively ensure food safety [28], as confirmed by the present study and previous research. However, there has been limited research on quantifying and demonstrating the relationship between food security emergency policies and food prices. While some existing studies have illustrated the relationship between food security policy and food price from the perspective of food policy reform [29], they have not analyzed this relationship in the context of responding to public health emergencies. Other studies have developed indicators, such as the monthly nominal rate of protection, an “express” indicator to reflect the real-time impact of food security emergency policy on grain prices [30,31,32], but have not considered the influence of food types and regional heterogeneity. This study differs from previous research in that it uses two indicators, the food price volatility index (*CV*) and food price increase rate (*ROC*), to quantitatively and visually analyze the relationship between China’s food security emergency policy and four kinds of food prices, in order to clarify the impact of food security emergency policy on food prices. Moreover, in the worst outbreak in Hubei province, food prices at the beginning of the lockdown (23 January to 27 January) showed sharp fluctuations, and the food price rise was bigger. However, with the unimpeded “green channel” (27 January, 29 January), the problem of food transportation caused by the lockdown policy was effectively solved, and the vegetable and meat prices decreased significantly. Secondly, with the cross-regional mobilization of “vegetable basket” products, such as the establishment of multi-regional “joint guarantee” mechanisms (17 February), the establishment of emergency agricultural product transfer stations, and the formation of cross-provincial supply guarantee networks (4 February), the main producing and consuming areas could be guaranteed to form a joint supply mechanism in time to ensure the food supply to the market. Then, relevant policies were issued to ensure the orderly resumption of market operation. Enterprises and volunteers distributed packages of “special price vegetable buns” and “special price meat buns”, mobile vegetable trucks entered communities in an orderly manner (17 February), and more daily necessity supply outlets were added, which further stabilized food prices. So, with these powerful food security emergency policies’ implementations in China, food price changes tended to be stable, and gradually dropped to the pre-lockdown level; this has been clearly demonstrated in our quantitative analysis.

In response to a public health emergency, ensuring the safety of staple foods is crucial to guarantee food security for all countries. Due to different food policy responses, fluctuations in staple food prices varied among countries during the COVID-19 pandemic. For example, while rice prices in the US and India increased differently [33,34], there was no significant change in China’s rice price [12]. Such variations are related to the implementation of food security policies [35]. From the perspective of grain reserve management, the US adopts a system that combines national regulation with market regulation, including federal reserves, farmer reserves, and free reserves. The federal reserve is implemented by credit companies and plays an important role in market regulation. The purpose of farmer reserves is to maintain stable food prices and reduce national food storage costs. Farmers must implement the government’s food production plan and sign contracts with credit companies for management. Both the federal and farmer reserves aim to balance market supply and demand, while the purpose of free reserves is to generate profits. India’s staple food reserves mainly include operational reserves and strategic reserves. Operational reserves mainly supply food to targeted public distribution systems and other government welfare programs, while strategic reserves serve as a supplement to operational reserves, mainly to address supply shortages and price fluctuations caused by uncertainty factors such as food production reduction [36]. China’s grain reserves mainly include central and local reserves. The central reserve is used to guarantee the national supply of grain in response to major natural disasters and emergencies, while local reserves are mainly used to address regional supply and demand imbalances, emergencies, and residents’ emergency food rations [37]. In terms of policy implementation, the US has a highly developed market economy, and its staple food reserves and sales mainly rely on market regulation by enterprises. India’s staple food reserves and circulation are almost government-led, while in China, the central grain reserve rights belong to the central government, which is specifically operated by the China Grain Reserve Corporation; local grain reserves are owned by local governments. The government can play the coordinating role of regulating the market and stabilizing grain prices through the mechanism of coordination between central and local grain reserves. We can conclude that policy management modes and implementation methods vary greatly among different countries, which is also important for changes in staple food prices.

Last but not least, this paper takes COVID-19 as an example to research the relationship between food security emergency policies and food prices from the perspective of coping with emergency risk shocks. However, in the research process, other random variables cannot be controlled when setting the model. There are other factors related to the volatility and changes in food prices, and other events or random effects will occur when the policies take effect. In addition, COVID-19 is an exceptional case of emergent risk, and future global scenarios (such as climate change, environmental sustainability, biodiversity losses, and price volatility) will alter the impact of weather on crop yields, thereby becoming a fundamental determinant of agricultural productivity and food insecurity [38,39,40]. Generally, shocks such as COVID-19 cannot be predicted in advance, and the impact of COVID-19 on food prices is uncertain [41]. Therefore, it is necessary to conduct a risk analysis on agricultural production first, regard weather shocks as random variables [42], and further analyze their impact on the distribution of crop yield distribution and price trend changes. However, it is challenging to estimate the distribution of crop yields. Because adverse risk shocks are first located in the lower tail of yield distributions, a simple mean variance analysis is not sufficient to evaluate the effects of possible crop failure [42,43,44]. Second, genetic selection and improved management have affected crop yield distributions, both across crops and over time. Third, crop yields vary across space because of agro-climatic conditions, and soil fertility changes across locations [45,46]. Establishing linkages between spatial and temporal agricultural risks and food insecurity remains difficult. In this analysis, we did not consider a risk analysis, and could not determine the spatial distribution of crop yields. Moreover, we did not predict whether the price trend would affect the farmers’ incomes overall. We considered COVID-19 as a known risk shock and analyzed its impact on food prices, which inevitably had certain limitations.

## 5. Conclusions

This study provides a scientific basis for improving the ability of the food system to cope with emergency risks from the perspective of food security policies. In the future, policy-makers can formulate response policies based on food types (including perishable foods, short-term stored food, and long-term stored food) in response to emergencies, which can effectively guarantee the supply of various foods in the context of public health emergencies. Through the joint supply mechanism between the main producing areas and main consuming areas, emergency policies for differentiated food supplies in producing and consuming areas can be formulated, thus improving the supply capacity of production materials in main producing areas, and the food circulation capacity in main consuming areas; this will effectively strengthen the food supply in both producing and consuming areas, which is of great significance for dealing with public health emergencies.

The impact of COVID-19 on the global food system is significant, and countries are continuously improving policy mechanisms to enhance the resilience of their food systems. The study of food security emergency policies and food prices in this paper will help to improve food supply capacity, stabilize food prices, ensure food safety, and improve the resilience of the food system to resist and cope with sudden risks from the perspective of policy security, so as to provide guidance, using China’s experience, for improving the global food security policy system.

Transport policies are very important to ensuring food supply. In the future, when formulating international food policies, we should open up trade and transportation corridors between main food-exporting countries and food-importing countries, especially when responding to public health emergencies. We should also open up green corridors for international transport in a timely manner, as this is conducive to ensuring global food security.

Finally, we must clearly understand that different countries have adopted various food emergency security policies [30] and used trade policy instruments [47] in response to the current public health emergency. In the future, more attention should be paid to the relationship between food security emergency policies and food prices in different countries around the world. Trade policy instruments can be used to further integrate global food security emergency policies and conduct a fitting analysis between global food security emergency policies and food prices to draw more comprehensive conclusions. The global food security policy system and the capacity of the global food system to respond to public health emergencies can always be improved. Furthermore, international experiences should be summarized through international research, and staple food reserves and management mechanisms around the world should be improved; these actions will help countries to avoid food crises caused by fluctuations in staple food prices that occur in response public health emergencies.

## Figures and Tables

**Table 1:** Lockdown time in all provinces.

Province Name	Start Time	End Time
Beijing	24 January 2020	5 March 2020
Shanghai	24 January 2020	5 March 2020
Guangdong	23 January 2020	5 March 2020
Hubei	23 January 2020	8 April 2020
Shandong	24 January 2020	5 March 2020
Henan	25 January 2020	5 March 2020

**Table 2:** Main food security emergency policies in China during the lockdown.

Time	Food Security Emergency Policy Name	Policy Type	Issuing Department
23 January 2020	Urgent notice on the control of transport vehicles entering and leaving Wuhan, sparing no efforts in epidemic prevention and control	Food transport	Ministry of Transport, China
27 January 2020	A notice on the recent work plan for the prevention and control of the novel coronavirus pneumonia infection	Food transport	National Health Commission, China
29 January 2020	Urgent notice on coordinating epidemic prevention and control and ensuring transportation	Food transport	Ministry of Transport, China
31 January 2020	The Ministry of Commerce of the National Development and Reform Commission will further provide comprehensive support for the transportation of daily necessities	Food transport	National Development and Reform Commission, China
1 February 2020	Urgent notice on ensuring the smooth passage of vehicles transporting emergency materials for epidemic prevention and control	Food transport	Ministry of Transport, China
4 February 2020	Urgent notice on ensuring the normal circulation order of agricultural products and means of agricultural production in the vegetable basket	Food supply	Ministry of Agriculture and Rural Affairs, China
5 February 2020	Notice on maintaining the normal production and marketing order of animal husbandry and ensuring the supply of meat, egg and milk markets	Meat supply	Ministry of Agriculture and Rural Affairs, China
6 February 2020	Notice the docking, transportation, insurance, and supply of living materials during the epidemic prevention and control period	Food supply	Ministry of Commerce, China
8 February 2020	The public security traffic control department implements the “emergency handling” service for vehicle license plates, efforts to ensure convenient transportation of epidemic prevention and control materials and daily necessities	Food transport	Ministry of Transport, China
12 February 2020	Circular of the State Council on the joint control mechanism for novel coronavirus infection on consolidating the “vegetable basket” mayor responsibility system and ensuring stable production and supply of agricultural products	Food supply	The State Council, China
15 February 2020	Urgent notice on solving the current practical difficulties and accelerating the resumption of production of the farming industry	Meat supply	Ministry of Agriculture and Rural Affairs, China
17 February 2020	A circular on further improving the joint contribution and cooperation mechanism of nine provinces during the prevention and control of the novel coronavirus epidemic	Food supply	Ministry of Commerce, China
21 February 2020	Circular on coordinating and ensuring the supply of daily necessities	Food supply	Ministry of Commerce, China
25 February 2020	A circular on promoting typical practices to ensure the supply of daily necessities during the epidemic prevention and control period	Food supply	Ministry of Commerce, China

**Table 3:** Impact of lockdown policies on food prices (DID results).

City	Food Price	Coefficient (α_3_)	Standard Error	Significance Levels	R^2^
Beijing	CP	0.2927	0.039	***	0.729
	PP	1.9719	0.214	***	0.971
	FP	0.0079	0.003	**	0.969
	RP	0.0053	0.001	***	0.514
Shanghai	CP	0.1663	0.040	***	0.536
	PP	1.8001	0.240	***	0.967
	FP	0.0467	0.001	***	0.957
	RP	−0.002	0.002	NO	0.749
Guangdong	CP	0.1798	0.057	**	0.377
	PP	NA	NA	NA	NA
	FP	NA	NA	NA	NA
	RP	NA	NA	NA	NA
Hubei	CP	0.4172	0.041	***	0.572
	PP	2.0492	0.198	***	0.931
	FP	0.0290	0.007	***	0.101
	RP	0.0111	0.003	***	0.456
Shandong	CP	0.5169	0.044	***	0.548
	PP	1.7032	0.223	***	0.978
	FP	0.0042	0.006	NO	0.427
	RP	0.0221	0.019	NO	0.139
Henan	CP	0.4186	0.029	***	0.648
	PP	2.1017	0.180	***	0.982
	FP	0.0034	0.004	NO	0.113
	RP	0.0048	0.004	NO	0.013

Note: ** and *** symbolize that the coefficients pass 5% and 1% significance levels, respectively. R^2^ represents the goodness-of-fit of the model. NO indicates that the coefficient does not pass the significance test. NA indicates that the data are missing. CP is Chinese cabbage price. PP is pork price. FP is flour price. RP is rice price. FP and RP are staple food prices.

## Data Availability

The lockdown time data in all provinces come from https://baijiahao.baidu.com/s?id=1665477599735122526&wfr=spider&for=pc (accessed on 1 October 2022). The food policies data come from http://www.gov.cn/zhengce/index.htm (accessed on 10 August 2022). Chinese cabbage price data in Beijing, Shanghai, Hubei and Guangdong are available from Ministry of Agriculture and Rural Affairs. Other food price data are available from the Wind database.
